# Lack of vimentin impairs endothelial differentiation of embryonic stem cells

**DOI:** 10.1038/srep30814

**Published:** 2016-08-02

**Authors:** Liana C. Boraas, Tabassum Ahsan

**Affiliations:** 1 Department of Biomedical Engineering, Tulane University, New Orleans, LA, USA.

## Abstract

The cytoskeletal filament vimentin is inherent to the endothelial phenotype and is critical for the proper function of endothelial cells in adult mice. It is unclear, however, if the presence of vimentin is necessary during differentiation to the endothelial phenotype. Here we evaluated gene and protein expression of differentiating wild type embryonic stem cells (WT ESCs) and vimentin knockout embryonic stem cells (VIM −/− ESCs) using embryoid bodies (EBs) formed from both cell types. Over seven days of differentiation VIM −/− EBs had altered morphology compared to WT EBs, with a rippled outer surface and a smaller size due to decreased proliferation. Gene expression of pluripotency markers decreased similarly for EBs of both cell types; however, VIM −/− EBs had impaired differentiation towards the endothelial phenotype. This was quantified with decreased expression of markers along the specification pathway, specifically the early mesodermal marker *Brachy-T*, the lateral plate mesodermal marker FLK1, and the endothelial-specific markers TIE2, PECAM, and VE-CADHERIN. Taken together, these results indicate that the absence of vimentin impairs spontaneous differentiation of ESCs to the endothelial phenotype *in vitro.*

Vascular endothelial cells are important for tissue engineering and regenerative medicine therapies designed to treat vascular pathologies. Applications include the endothelialization of vascular grafts to prevent thrombosis, vascularization of tissue engineered organs, or augmentation of vessel growth in ischemic tissue after injury. Such therapies, however, require large numbers of endothelial cells, which are difficult to obtain as primary cells or with stable phenotypic expansion. Pluripotent stem cells, including embryonic stem cells, have the capacity to self-renew and differentiate to all phenotypes in the body and are therefore an attractive source for cell-based therapies.

For the success of vascular therapies, stem cell-derived endothelial cells must recapitulate the essential attributes of mature endothelial cells. During normal physiology, endothelial cells (ECs) *in vivo* are exposed to blood flow-induced shear stress. A proper response to this mechanical cue is pivotal for maintaining the physiologic endothelial phenotype. Nitric oxide and sodium regulation, as well as cytoskeletal alignment, are regulated *in vivo* by blood flow^1^. Multiple cytoskeletal proteins are also remodeled *in vitro* as part of the endothelial mechanoresponse. For example, actin stress fibers that span the cell realign in the direction of flow^2^ and the network of vimentin molecules undergo micrometer and nanometer level displacements^3^,^4^ in normal ECs exposed to shear stress. A robust cytoskeletal infrastructure is therefore an inherent trait of functional ECs.

The cytoskeleton network is composed of three categories of structural proteins: microtubules, microfilaments, and intermediate filaments. Vimentin, an intermediate filament with a diameter of approximately 10 nm, is thought to provide mechanical integrity and structural support to cells^5^. While expressed in a variety of mesenchymal cell types, vimentin is a critical player in the physiologic endothelial mechanoresponse and is inherent to the endothelial phenotype^4^,^6^. In knockout animals, the loss of vimentin results in viable mice but has been implicated in pathological vascular function. Vimentin −/− mice compared to the wild type have been observed to have a smaller carotid artery^7^, decreased flow-induced arterial dilation^7^, delayed arterial remodeling^8^, and increased permeability of the endothelial barrier^9^. Thus, the presence of vimentin is necessary for proper endothelial function in adult mice.

Vimentin is inherent to fully differentiated ECs, yet it is unclear if the presence of vimentin is necessary during *in vitro* differentiation. Here we formed embryoid bodies from both wild type embryonic stem cells and vimentin knockout embryonic stem cells to study differentiation towards the endothelial phenotype. Over 7 days of spontaneous differentiation, the wild type cells increased expression of endothelial specific markers by 4-90X, which was a ~5-fold greater change than that observed with the vimentin knockout cells. Thus, the lack of vimentin in embryonic stem cells resulted in impaired endothelial differentiation *in vitro*.

## Results

### Pluripotency of Vimentin −/− Stem Cells

**Figure 1:**
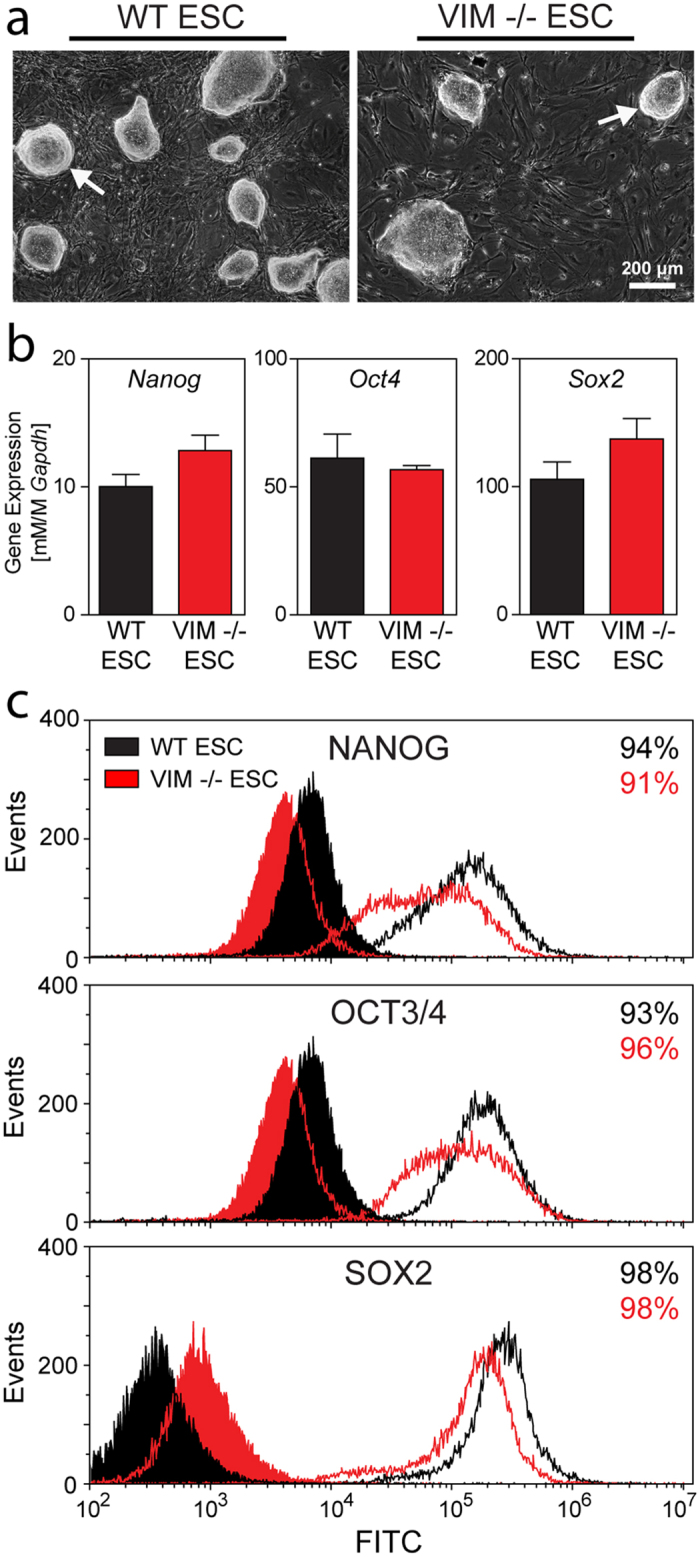
Expression of pluripotency markers are similar between WT ESCs and VIM −/− ESCs. (**a**) Representative phase images are shown of WT ESCs and VIM −/− ESCs cultured on MEF feeder layers. Arrows indicate refractive edges of cell colonies. Scale bar represents 200 μm. (**b**) Gene expression of *Nanog, Oct4*, and *Sox2 (*all normalized to *Gapdh*) are shown for both cell types. Data are presented as mean ± SEM (n = 3). (**c**) Flow cytometry analysis for both cell types of NANOG, OCT3/4, and SOX2 are shown. Shaded histograms are for staining (secondary antibody-only) controls. Values listed are for the percentage of cells within the population considered to be positive. WT ESCs are represented in black and VIM −/− ESCs are in red.

Vimentin knockout embryonic stem cell (VIM −/− ESC) and wild type embryonic stem cell (WT ESC) samples were morphologically similar when cultured on feeder layers (Fig. 1a). Both cell types formed colonies with refractive edges containing tightly compact cells (Fig.1a arrows), typical of pluripotent stem cells. WT ESCs and VIM −/− ESCs were also evaluated for gene (Fig. 1b) and protein (Fig. 1c) expression of pluripotency markers. Gene expression levels of pluripotency markers (*Nanog, Oct4, Sox2*) were similar for both cell types with no significant differences detected for any of the three genes (*Nanog*: p = 0.158; *Oct4*: p = 0.558; *Sox2*: p = 0.233). Likewise, flow cytometry analysis revealed that both VIM −/− ESC and WT ESC populations had largely similar levels of protein expression of the three pluripotency markers. Compared to WT ESCs, VIM −/− ESCs had a wider distribution of expression intensities for NANOG and OCT3/4, though both populations were >90% positive compared to their negative controls. Both cell types were also 98% positive for SOX2 (compared to negative controls) and had similar expression profiles on the population level. Thus, both WT ESCs and VIM −/− ESCs had similar morphologies as well as similar expression levels of pluripotency markers. Taken together, these results indicate that both WT ESCs and VIM −/− ESCs have a similar state of pluripotency during *in vitro* culture.

### Embryoid Body Morphology and Proliferation

**Figure 2:**
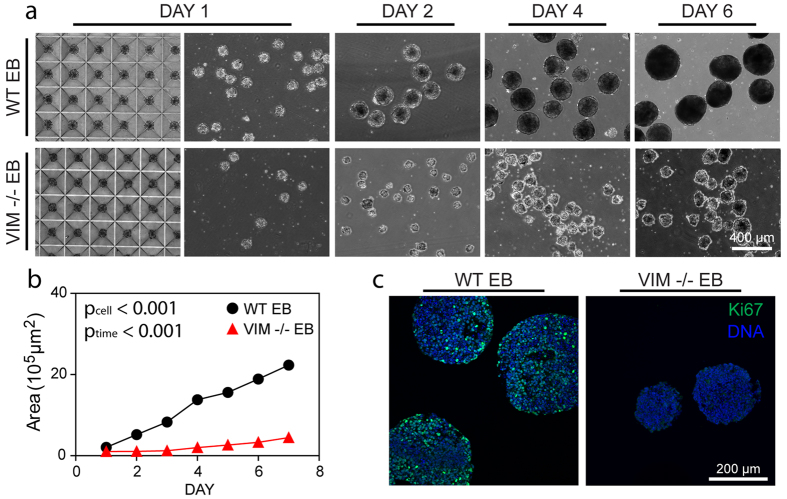
Growth of VIM −/− EBs is slower than that of WT EBs. (**a**) Phase images for WT EBs and VIM −/− EBs at Day 1 before (left image) and after removal (right image) from the microwells, as well as at Day 2, 4, 6 in suspension culture. All images are at the same magnification and the scale bar represents 400 μm. (**b**) Cross sectional areas for WT EBs and VIM −/− EBs were calculated from phase images (n = 50 EBs per group). (**c**) Immunohistochemical analysis indicates the proliferation marker Ki67 (green) with a nuclear counterstain (blue). Scale bar represents 200 μm.

Embryoid Bodies (EBs) were generated from either vimentin knockout or wild type embryonic stem cells to evaluate differences during spontaneous differentiation. VIM −/− ESCs failed to form EBs under standard rotary conditions (Supplementary Fig. S1). Consequently, physical aggregation with microwells was used to create EBs from VIM −/− ESCs. WT EBs were similarly generated to allow for direct comparison. After 1 day in the microwells, both wild type and vimentin knockout cells aggregated to form EBs (WT EBs and VIM −/− EBs, respectively) that remained intact upon removal from the microwells (Fig. 2a). VIM −/− EBs agglomerated under rotary culture (Supplementary Fig. S1), so all EBs were instead cultured under static conditions. Size analysis of phase images revealed that EBs generated from either cell type increased in size over the culture period (Fig. 2a,b; p_time_ < 0.001). Compared to WT EBs, however, VIM −/− EBs had markedly lower growth rates leading to smaller EBs (p_cell_ < 0.001 and p_cellxtime_ < 0.001). These findings were corroborated by immunohistochemical analysis of samples with Ki67, a nuclear marker of proliferation (Fig. 2c). While Day 6 WT EB samples had many cells that stained intensely for Ki67, time matched VIM −/− EBs had little to no detectible expression. Thus both cell types were able to form EBs under physical aggregation, though vimentin −/− cells proliferated less during differentiation and resulted in smaller embryoid bodies.

**Figure 3:**
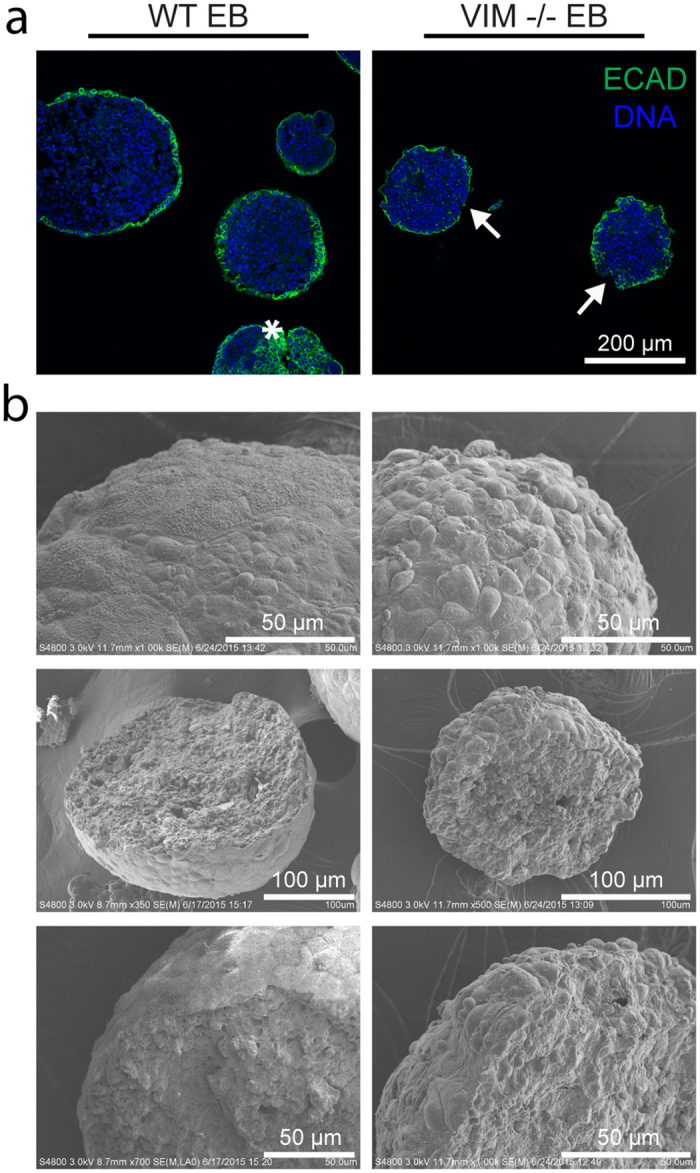
EB surface properties are distinct in VIM −/− EBs compared to WT EBs. (**a**) Histological sections were stained for ECAD protein (green) with a nuclear counterstain (blue). The star indicates staining within the interior of a WT EB and arrows indicate discontinuous ECAD expression along VIM −/− EB outer layers. All images are at the same magnification and the scale bar represents 200 μm. (**b**) SEM images were taken of both whole and fractured WT EBs and VIM −/− EBs. Length of scale bars is indicated in each image.

The morphological properties of the EBs from the different cell types were dissimilar. Phase images indicated that WT EBs established a smooth outer layer, while VIM −/− EBs had a less well defined border (Fig. 2a). Immunohistochemical analysis for the epithelial cell-cell adhesion molecule (ECAD) showed that WT EBs had some staining in the interior (Fig. 3, star), but predominantly had a continuous layer of expression at the periphery (Fig. 3). VIM −/− EBs also had expression at the periphery, however it was discontinuous (Fig. 3, arrow). Similarly, higher resolution SEM images of intact EBs showed that WT EBs had a smooth outer layer, while the surfaces of VIM −/− EBs were rippled due to more rounded cells. Images of fractured EBs, however, showed no apparent differences in cell organization in the interior of the EBs. These results indicate that WT EBs form a smooth tight outer layer, but that the VIM −/− EBs may have disrupted cell-cell junctions as indicated by decreased ECAD staining and a rippled outer surface.

### Pluripotency in Embryoid Bodies

**Figure 4:**
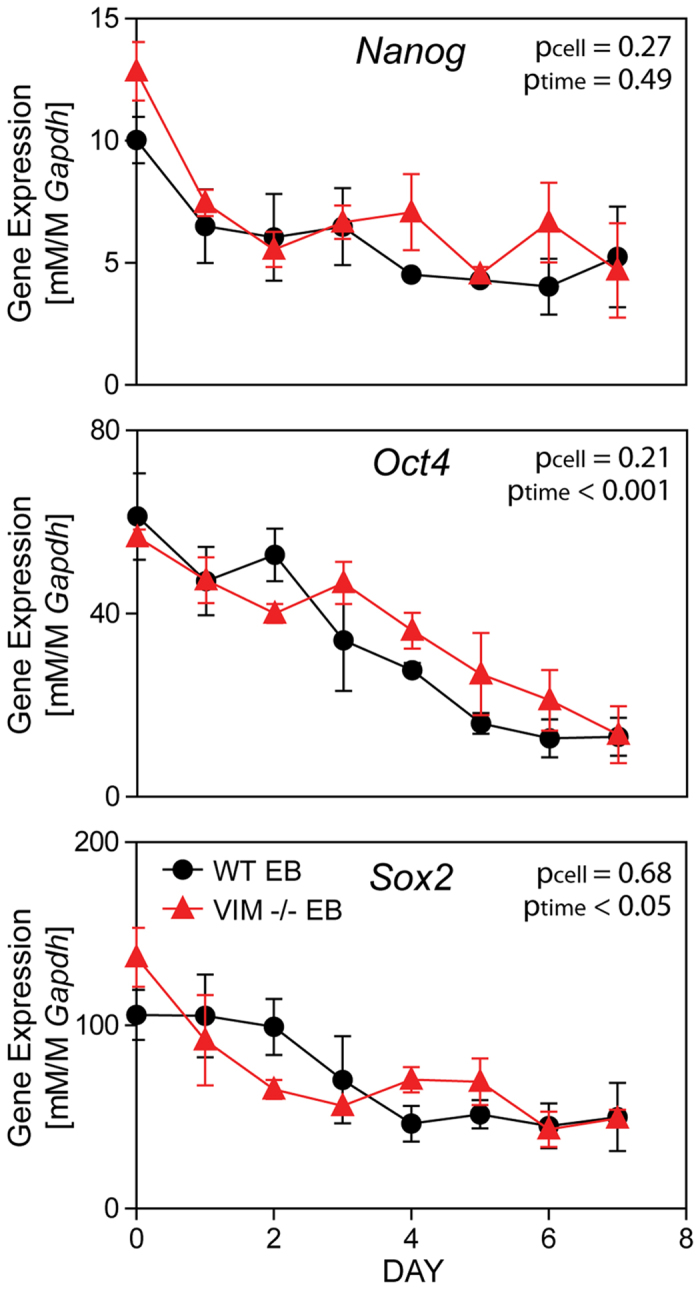
WT EBs and VIM −/− EBs similarly lose expression of pluripotency markers with differentiation. Gene expression of *Nanog, Oct4*, and *Sox2* (all normalized to *Gapdh*) are shown for WT EBs and VIM −/− EBs over 7 days of differentiation as EBs. Data presented as mean ± SEM (n = 3).

Change in pluripotency of WT EBs and VIM −/− EBs was evaluated by gene expression of *Nanog, Oct4*, and *Sox2* (Fig. 4). In the WT EBs, *Nanog* expression generally trended downward with differentiation from Day 0 to Day 7. Expression in VIM −/− EBs followed similar trends, with no detected difference between the two cell types. Similarly, expression of *Oct4* and *Sox2* in EBs of each cell type decreased significantly over time (*Oct4*: p_time_ < 0.001; *Sox2*: p_time_ < 0.05) with no differences observed between cell types. Thus, both wild type and vimentin −/− cells similarly lose expression of pluripotency markers during differentiation as embryoid bodies.

### Early Mesodermal Differentiation

**Figure 5:**
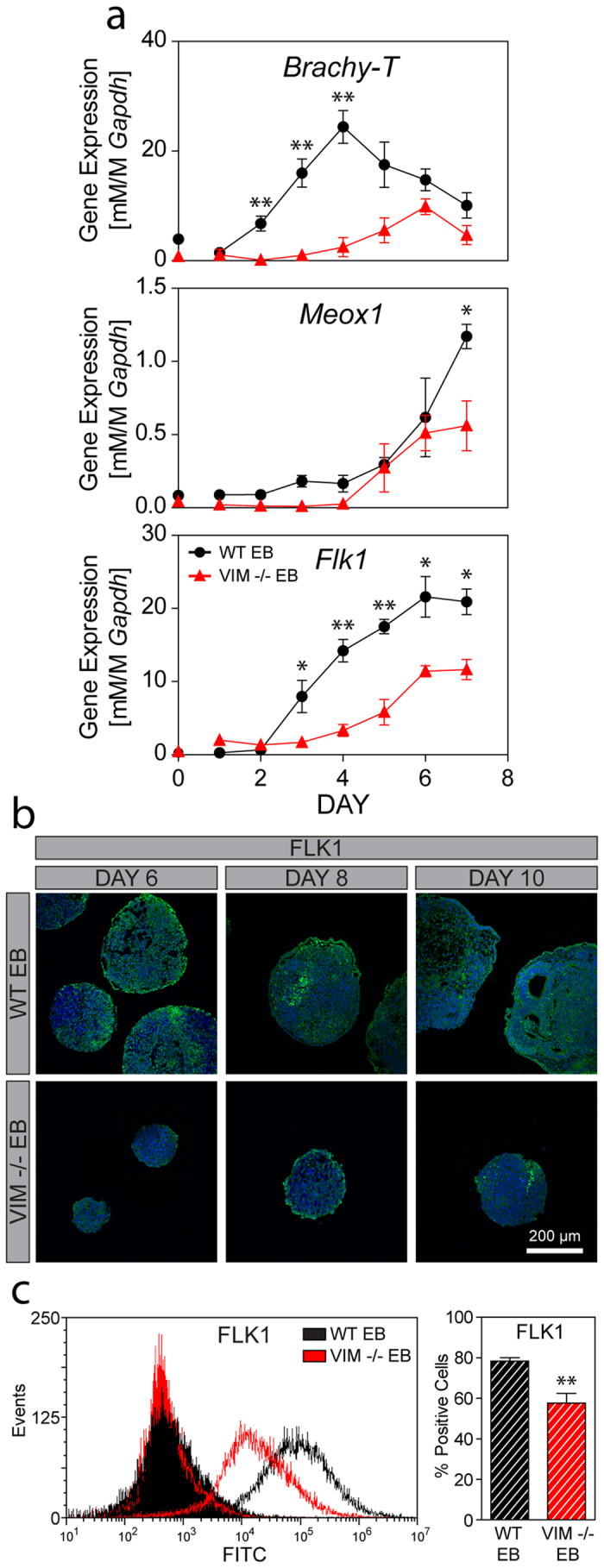
VIM −/− EBs have decreased mesodermal commitment compared to WT EBs. (**a**) Gene expression of *Brachy-T* (mesodermal commitment), as well as *Meox1* (paraxial mesoderm) and *Flk1* (lateral plate mesoderm) are shown for WT EBs and VIM −/− EBs over 7 days of differentiation (all normalized to *Gapdh*). (**b**) Immunohistochemical analysis of FLK1 protein expression (green) with a nuclear counterstain (blue) in EBs at Days 6, 8, and 10. All images were taken at the same magnification and the scale bar represents 200 μm. (**c**) A representative histogram of FLK1 protein expression is shown for WT EBs (black) and VIM −/− EBs (red), as well as their respective secondary antibody-only controls (shaded histograms). The bar graph shows the percentage of positive cells for each group at Day 10. Data are presented as mean ± SEM (n = 3 for gene expression; n = 4 for protein expression) with significant differences indicated using asterisks (*p < 0.05, **p < 0.01, ***p < 0.001).

Evaluation of early mesodermal commitment was evaluated with gene expression of *Brachy-T* (Fig. 5a). WT EBs expressed *Brachy-T* transiently with levels peaking at Day 4, which is consistent with previous findings^10^,^11^,^12^. Overall, *Brachy-T* expression in VIM −/− EBs was markedly and significantly (p_cell_ < 0.001) lower compared to the wild type samples throughout the culture period. In comparing each timepoint, post-hoc Tukey tests indicated that VIM −/− EBs had significantly (**p < 0.01) lower expression of *Brachy-T* compared to WT EBs specifically for Days 2 to 4. A slight elevation in expression at Days 5–7 was observed in VIM −/− EBs, but levels still remained low. Thus, overall VIM −/− EBs had a decreased mesodermal commitment over 7 days of differentiation compared to WT EBs.

Differentiation to specific mesodermal plates was evaluated with *Meox1* and *Flk1,* markers for the paraxial and lateral plate mesoderm, respectively (Fig. 5a). *Meox1* expression in EBs from each cell type had a similar and significant (p_time_ < 0.001) increase over 7 days of culture. Post hoc analysis did detect, however, a significantly higher expression in WT cells compared to VIM −/− at Day 7 (*p < 0.05). In evaluating *Flk1*, expression in WT EBs increased in a sigmoidal pattern to 80X of initial values over the 7 days of culture. Overall expression of *Flk1* in VIM −/− EBs was statistically (p_cell_ < 0.001) lower compared to those WT controls; initial *Flk1* expression levels were similar in both cell types until Day 3 after which levels in WT EBs increased significantly (*p < 0.05, **p < 0.01) more than in VIM −/− EBs. These large differences in *Flk1* gene expression were corroborated with visualization and quantification of protein expression (Fig. 5b,c). At Days 6, 8, and 10, WT EB samples all showed FLK1 protein expression both along the outer edge and in the interior of the EBs. Expression in the middle of the EBs was patchy with some areas of concentrated expression compared to regions with less visible staining. VIM −/− EBs, on the other hand, had very little overall FLK1 expression, with the limited expression observable at both the periphery and the interior. Flow cytometry analysis further revealed that protein expression after 10 days of differentiation in VIM−/− EBs was significantly lower than that in time matched WT EBs (p < 0.01: 58 ± 4.8% vs 78 ± 1.7%, respectively). Thus, both gene and protein analysis indicate that VIM −/− EBs compared to WT EBs have decreased specification to overall mesoderm and markedly impaired differentiation to the lateral plate mesoderm.

### Endothelial Differentiation

**Figure 6:**
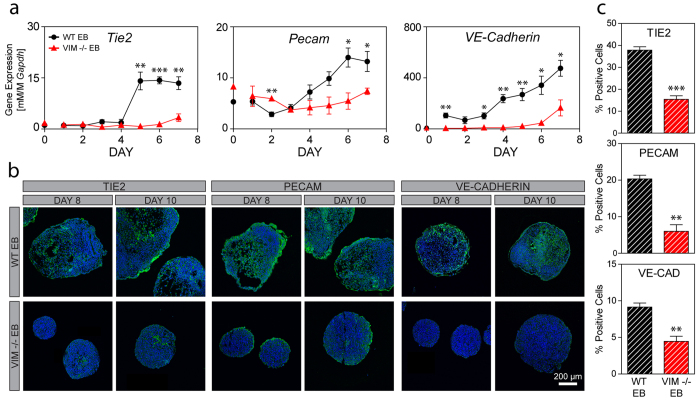
Endothelial differentiation is impaired in VIM −/− EBs compared to WT EBs. (**a**) Gene expression of *Tie2, Pecam*, and *VE-cadherin* are shown for WT EBs and VIM −/− EBs over 7 days of differentiation (all normalized to *Gapdh*). (**b**) Immunohistochemical analysis of TIE2, PECAM, and VE-CADHERIN protein expression (green) with a nuclear counterstain (blue) in EBs at Days 8 and 10. All images were taken at the same magnification and the scale bar represents 200 μm. (**c**) The bar graphs show the percentage of positive cells for TIE2, PECAM, and VE-CADHERIN at Day 10. Data are presented as mean ± SEM (n = 3 for gene expression; n = 4 for protein expression) with significant differences indicated using asterisks (*p<0.05, **p<0.01, ***p<0.001).

Endothelial differentiation, which arises from the lateral plate mesoderm, is robustly observed in embryoid bodies during spontaneous differentiation. Over 7 days of differentiation, gene expression in WT EBs increased significantly (p < 0.001 for all) for the endothelial markers *Tie2, Pecam*, and *VE-Cadherin* by 14X, 4X, and 90X, respectively (Fig. 6a). In WT EBs, *Tie2* and *Pecam* expression, similar to *Flk1* expression, increased in a sigmoidal pattern with relatively low initial levels followed by markedly higher subsequent levels. *Tie2* expression for Days 5–7 were similar to each other but significantly (p < 0.001) higher than the expression for all of the initial 4 days. Similarly for *Pecam*, gene expression for both Day 6 and 7 were significantly (p < 0.05) higher than all levels during the initial 3 days. In contrast, gene expression of *Tie2* and *Pecam* in the VIM −/− EBs was not sigmoidal but instead remained relatively low throughout differentiation. *Tie2* gene expression increased significantly (p < 0.05) over time but never rose above the initial levels found in the WT EBs and no significant differences were detected for *Pecam* expression over time in VIM −/− EBs. Gene expression of *VE-Cadherin*, a marker of the mature endothelial phenotype, increased steadily over 7 days of differentiation in WT EBs. Expression levels in VIM −/− EBs were consistently lower compared to time matched wild type controls and reached only 35% of the control value at Day 7. Overall, there was a statistically significant (p_cell_ < 0.001) difference in gene expression between the cell phenotypes for all three endothelial markers.

Expression at the protein level for these endothelial differentiation markers was evaluated in histology samples of Day 6, 8, and 10 EBs and with flow cytometry at Day 10. At Day 6 (Supplementary Fig. S4), WT EBs had some expression of the early endothelial marker TIE2, but almost no expression of PECAM or VE-CADHERIN. By Days 8 and 10 (Fig. 6b), however, WT EBs had acquired robust expression of all three endothelial proteins throughout large regions of the cell clusters. In contrast, VIM −/− EBs had very little expression of endothelial proteins over the 10 days of differentiation. In those samples at Day 8 and 10, a modest amount of TIE2 expression was visible in the interior of some EBs, while traces of PECAM were observed at the outer edges of the cell clusters. VE-CADHERIN, a later protein marker of the endothelial phenotype, was not detected in any of the VIM −/− EBs. Furthermore, quantitation using flow cytometry of Day 10 EBs revealed a significantly lower percentage of cells positively expressing TIE2 in VIM −/− EBs compared to WT EBs (15 ± 1.6% compared to 38 ± 1.5%, p < 0.001). Similarly, VIM −/− EBs, compared to WT EBs, had a reduced percentage of cells expressing PECAM (6 ± 1.8% compared to 20 ± 1.0%, p < 0.01) and VE-CADHERIN (4 ± 0.7% compared to 9 ± 0.6%, p < 0.01). Thus VIM −/− EBs, in contrast to WT EBs, have significantly lower gene or protein expression of endothelial markers.

## Discussion

Vimentin knockout embryonic stem cells (VIM −/− ESCs) were similar in pluripotency to wild type embryonic stem cells (WT ESCs), yet their capacity to form and differentiate as embryoid bodies (EBs) was markedly impaired. Gene and protein expression of pluripotency markers in undifferentiated cells were similar for both phenotypes. For the VIM −/− ESCs, however, EB formation required an initial 24 hours of physical aggregation prior to static suspension culture. Over seven days of differentiation, the VIM −/− EBs displayed an altered morphology compared to wild type controls, with a smaller size due to decreased proliferation and a rippled outer surface with disrupted ECAD expression. While gene expression of pluripotency markers decreased similarly for EBs of both cell types, VIM −/− EBs had impaired differentiation towards the endothelial phenotype. This was observed with decreased expression of markers along the specification pathway, specifically the early mesodermal marker *Brachy-T*, the lateral plate mesodermal marker FLK1, and the endothelial-specific markers TIE2, PECAM, and VE-CADHERIN. Quantitatively, over 7 days of spontaneous differentiation the wild type cells increased expression of endothelial specific markers by 4-90X, which was a ~5-fold greater change than that observed with the vimentin knockout cells. These same effects were not observed in differentiation to other mesodermal phenotypes, including cardiovascular and orthopedic, which also express vimentin (Supplementary Fig. S3). Thus, these results indicate that the absence of vimentin impairs differentiation in the embryoid body model of ESCs to the endothelial phenotype.

Knockout mice lacking vimentin initially were deemed phenotypically normal^13^, however subsequent analysis of these animals has shown differences in vascular remodeling and wound healing processes. Vimentin −/− mice have delayed arterial remodeling^8^ and decreased flow-induced arterial dilation^7^. Specifically in the endothelial cells of these knockout mice, vimentin has been shown to be important in regard to the integrity of the vascular endothelium^14^. Similar results were found in an *in vitro* study where disruption of vimentin in an endothelial cell monolayer led to increased permeability^9^. While these previous studies reveal that the absence of vimentin can affect vascular function, the studies performed here show that vimentin is critical in differentiation towards the endothelial phenotype *in vitro*.

This study found that cells lacking vimentin had impaired spontaneous differentiation *in vitro* toward the endothelial phenotype. Endothelial differentiation in the absence of vimentin is possible, however, during development^13^ and teratoma formation^15^ *in vivo*. Such observations indicate, that the complex tissue environment *in vivo* includes a redundancy for the function of vimentin that may not be recapitulated in isolated cells *in vitro*. Endothelial differentiation *in vivo* has been shown to be influenced by chemical signaling, including the VEGF^16^,^17^, BMP^16^, and Wnt^17^ pathways, as well as physical factors such as cell-cell contact^17^, cell-matrix adhesion^16^,^18^, and hemodynamic forces^19^. Additional directed differentiation studies *in vitro* may be useful to identify the additional chemical or physical factors that compensate for the absence of vimentin.

The lack of vimentin leads to decreased contractile capabilities and migration, as has been shown through decreased migration of fibroblasts^20^, reduced compaction of collagen gels^20^, and impaired wound healing in vimentin −/− mice^21^,^22^. Intracellular contraction that results in migration is also important during development, including the epithelial to mesenchymal transition (EMT) of gastrulation^23^. Cells in the inner cell mass, from which pluripotent ESCs are derived, differentiate as they form the ectodermal, mesodermal, and endodermal germ layers. In particular, cells of the mesoderm need the cytoskeletal infrastructure to become motile in order to penetrate the primitive streak on the ectoderm and migrate to fill the space below. This is consistent with our own previous *in vitro* studies in which we found that pluripotent ESCs have little cytoskeleton^24^ and that cytoskeletal expression increases markedly with differentiation^24^,^25^. Here we have begun to understand the role of the cytoskeleton in differentiation in that vimentin is pivotal to differentiate ESCs to the mesodermal lineage and the endothelial phenotype *in vitro*.

Vimentin has also been implicated in cellular interactions with the external microenvironment. We found that VIM −/− cells have altered cell-cell interactions compared to wild type controls, as evidenced by a failure to aggregate to form clusters during suspension-induced EB formation, as well as disrupted ECAD expression and a rippled surface in the outer layer of physically-aggregated EBs. Studies by others have shown that focal contacts in vimentin knockout cells have a disrupted architecture^20^, are of smaller size^26^, and have destabilized adhesion to the extracellular matrix^18^. It has also been shown that vimentin has a functional role in adhesive strength through its interactions with plectin^27^, a cytoplasmic cross-linker connecting intermediate filaments to microtubules, microfilaments, and membrane adhesion proteins^28^. These studies, taken together with the findings presented here, provide further evidence that vimentin has a role in the transmembrane protein complexes that regulate adhesion to adjacent cells and matrix proteins.

Mechanical forces have been shown to regulate stem cell fate including the process of differentiation. For example, our group has shown that embryonic stem cells (ESCs) exposed to fluid shear stress differentiate toward the mesodermal lineage^12^ and specifically to the endothelial phenotype^29^. The cytoskeleton is known to regulate the cellular response to mechanical forces and remodel during differentiation^25^ and dedifferentiation^24^. While these studies investigated the role of vimentin during spontaneous differentiation, what has yet to be determined is the role of vimentin during directed differentiation in response to mechanical cues.

Vimentin is a structural protein that may also play a role in intracellular signaling. Small precursor subunits^30^,^31^ of vimentin have been shown to travel intracellularly along microtubules with the aid of kinesin^30^,^32^ and dynein^33^. These subunits, in addition to integrating with the overall vimentin network, can also aid in signaling processes^34^. Specifically, vimentin subunits may directly interact with ERK to modulate signaling during EMT^35^ and to transport phosphorylated ERK1/2 to the nucleus of cells after nerve injury^36^. Thus, it is possible that vimentin can modulate differentiation by regulating biochemical signaling cascades.

Vimentin may also play a role in cellular mechanics and the mechanoresponse directly or through its interaction with other cytoskeletal proteins. Vimentin is known to link to both microtubules (MTs) and microfilaments (MFs)^37^. Furthermore, the tail end of vimentin can link directly to MFs or to actin containing structures^38^,^39^. These interactions may account for observed alterations in MT patterning and polarity in vimentin knockout fibroblasts^40^. Conversely, the vimentin network is disrupted when MT or MF networks depolymerize^41^. These changes in cytoskeletal organization lead to changes in cellular mechanical properties. In the case of vimentin, its absence has been shown to increase deformability^42^ and shear modulus^43^, as well as increase cell viscosity and decrease resistance to compression^44^. Hence, the absence of vimentin leads to changes in cytoskeletal organization which alters the overall mechanical properties of the cell and therefore its mechanoresponse.

Vimentin is an intermediate filament long known to be expressed in endothelial cells. Cellular therapies designed to treat vascular pathologies rely on large numbers of endothelial cells. The use of highly proliferative stem cells as a cell source requires a thorough understanding of the differentiation process. Using knockout ESCs, we found that the absence of vimentin impairs spontaneous endothelial differentiation *in vitro* and have furthered our understanding of the regulators of differentiation. This type of study is necessary for the rational design of efficient differentiation protocols to generate clinically-relevant numbers of cells for tissue engineering and regenerative medicine applications.

## Methods

### Embryonic Stem Cell Culture

Vimentin knockout mouse embryonic stem cells (VIM −/− ESCs; strain C57BL/6; Vim_AF3 from the KOMP Repository) and wild type mouse embryonic stem cells (WT ESCs; strain 129; ESD3 cells from ATCC™) were expanded as previously described^12^,^25^,^29^,^45^. Briefly, ESCs were initially expanded on a mitotically inactivated feeder layer and then expanded for at least one passage on gelatin-coated plastic in culture medium, which consisted of Dulbecco’s Modification of Eagles Medium supplemented with 15% ES-qualified fetal bovine serum (Invitrogen), 2 mM L-glutamine, 0.1 mM non-essential amino acids, 0.1 mM beta-mercaptoethanol, 1,000 U/ml Leukemia Inhibitory Factor (LIF; ESGRO^®^ from EMD Millipore) and antibiotics.

### Embryoid Body Differentiation

WT ESCs and VIM −/− ESCs were spontaneously differentiated as embryoid bodies (EBs) in 3D suspension culture, referenced in these studies as WT EBs and VIM −/− EBs, respectively. VIM −/− ESCs did not spontaneously aggregate in suspension culture (Supplementary Fig. S1), requiring that EBs be generated using commercially available microwells (AggreWell™) that induce physical aggregation^46^,^47^. Both types of ESCs were dissociated and seeded at a density of 800 cells/microwell for 24 hours. EBs were then transferred to agar-coated non-tissue culture dishes and maintained for up to 10 days in medium without LIF. Medium and dishes were changed using gravity separation every 24 hours after the third day. Vimentin expression remained low in VIM −/− EBs over the culture duration, validating the knockout of vimentin expression (Supplementary Fig. S2).

### Morphological Assessment

Phase contrast microscopy and scanning electron microscopy (SEM) were used to determine the morphology of pluripotent cell colonies and EBs. EB size was evaluated by cross sectional areas as determined using ImageJ software and phase images of EBs generated from each cell type (n = 50 EBs per group). Details of EB topology were visualized using SEM. EBs for this analysis were fixed in 2.5% glutaraldehyde and 1% osmium tetroxide/0.1 sodium cacodylate solution (Sigma Aldrich^®^). Samples were then fractured, dehydrated, sputter-coated with carbon, and assessed on a Hitachi 4800 system.

### Gene Expression

Samples were evaluated for gene expression as described previously^12^. For each sample, RNA was isolated (Qiagen), converted into cDNA (Invitrogen), and analyzed using standard real-time PCR with SYBR^®^ Green on a StepOnePlus™ PCR System (Applied Biosystems). Primers were designed to assess pluripotency (Homeobox Transcription Factor Nanog: *Nanog*; Octamer-Binding Protein: *Oct4*; Sex Determining Region y-Box 2: *Sox2*), mesodermal commitment (T-homeobox domain: *Brachy-T*), mesodermal differentiation (mesenchyme homeobox 1: *Meox1*; vascular endothelial growth factor receptor 2: *Flk1*) and endothelial differentiation (Tyrosine Kinase: *Tie2*; Platelet/Endothelial Cell Adhesion Molecule: *Pecam*; Cadherin 5, Type 2 Vascular Cadherin: *VE-Cadherin*). Gene expression levels were determined using standard curves and reported normalized to glyceraldehyde-3-phosphate dehydrogenase (*Gapdh*).

### Protein Expression

Standard protocols for immunohistochemistry and flow cytometry were used to determine protein expression as described previously^12^,^48^. EBs were fixed in 4% formaldehyde, paraffin embedded, and sectioned (6 μm slices). Sample sections were deparaffinized, heat treated for antigen retrieval, blocked for non-specific binding with serum, and then stained with primary and secondary antibodies, as well as HOECHST 33258 as a nuclear counterstain. Primary antibodies used were specific for Ki67 (Abcam), α-E-Cadherin (ECAD; R&D Systems^®^), FLK1 (Santa Cruz), TIE2 (PE-conjugated, Abcam), PECAM (Santa Cruz), VE-CADHERIN (Santa Cruz). Secondary antibodies were conjugated with AF488 (Molecular Probes, Eugene, OR) and samples were visualized with a Nikon A1 confocal microscope.

Protein expression was quantified using flow cytometry. EBs were dissociated using StemPro^®^ Accutase^®^ (Life Technologies) and mechanical trituration. Cell solutions were then fixed in 4% formaldehyde, permeabilized using 0.5% triton-X (Sigma), blocked with serum, and stained with primary and secondary antibodies (listed above). Primary antibodies used were specific for NANOG (Abcam), OCT3/4 (Santa Cruz), SOX2 (FITC-conjugated, eBiosciences), FLK1 (Santa Cruz), TIE2 (PE-conjugated, Abcam), PECAM (Santa Cruz), VE-CADHERIN (Santa Cruz). For each sample, the cells were considered positive if expression was above 99% of the matched secondary only control populations. An Attune Acoustic Focusing Cytometer (Applied Biosystems) was used for fluorescence detection.

### Statistical Analysis

Quantitative data are represented as mean ± SEM for n = 3 independent trials for gene expression analysis, n = 50 for EB size quantification analysis, and n = 4 for protein expression analysis. Direct comparisons between WT ESCs and VIM −/− ESCs were analyzed using two-tailed Student’s t-tests. The kinetic data was analyzed with a 2-way ANOVA using a post hoc Tukey test as appropriate for comparisons on time, cell type, and their interaction. Differences were considered statistically significant for p-values < 0.05.

## Additional Information

**How to cite this article**: Boraas, L. C. and Ahsan, T. Lack of vimentin impairs endothelial differentiation of embryonic stem cells. *Sci. Rep.* **6**, 30814; doi: 10.1038/srep30814 (2016).

## Supplementary Material

Supplementary Information
