# Supplementary material for: Lack of vimentin impairs endothelial differentiation of embryonic stem cells
Source: Sci Rep. 2016 Aug 2;6:30814. doi: 10.1038/srep30814 (PMC4969593; doi:10.1038/srep30814)
Supplement: Supplementary Information [file srep30814-s1.pdf]

LACK OF VIMENTIN IMPAIRS ENDOTHELIAL DIFFERENTIATION OF EMBRYONIC STEM CELLS

Liana C. Boraas & Tabassum Ahsan*

Department of Biomedical Engineering
Tulane University
New Orleans, LA

*Corresponding author
Tabassum Ahsan
Tulane University
Department of Biomedical Engineering
500 Lindy Boggs Center
New Orleans, LA USA 70118
PHONE: (504) 988-6955
EMAIL: tahsan@tulane.edu

Supplementary Figure S1

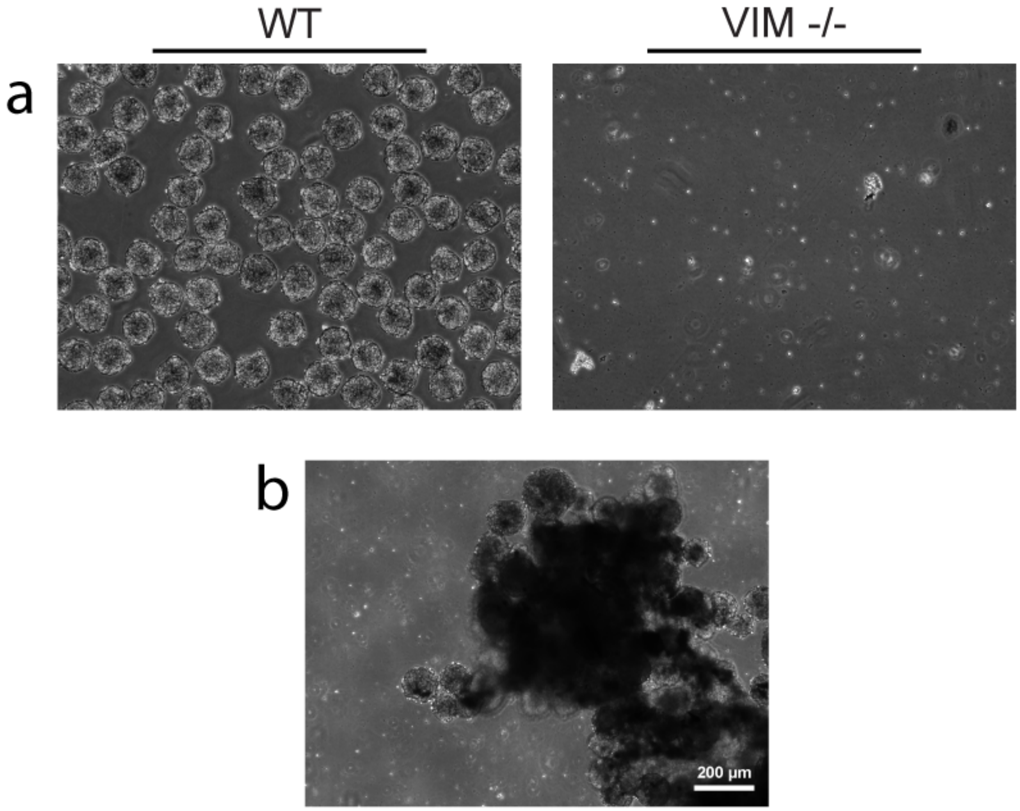

Supplementary Fig. S1. Formation of EBs from VIM -/- ESCs requires physical aggregation in microwells and subsequent culture under static conditions. (a) WT ESCs plated at 0.5x10^6 in 10 mls of differentiation medium and placed on a rotary shaker at 40 RPM cluster to form embryoid bodies. VIM -/- ESCs under similar conditions failed to cluster to form any visible EBs. **(b)** Microwells can be used to physically aggregate VIM -/- ESCs to form EBs, but subsequent culture under rotary conditions forms large agglomerations. All phase images were taken at the same magnification and the scale bar represents 200 μm.

Supplementary Figure S2

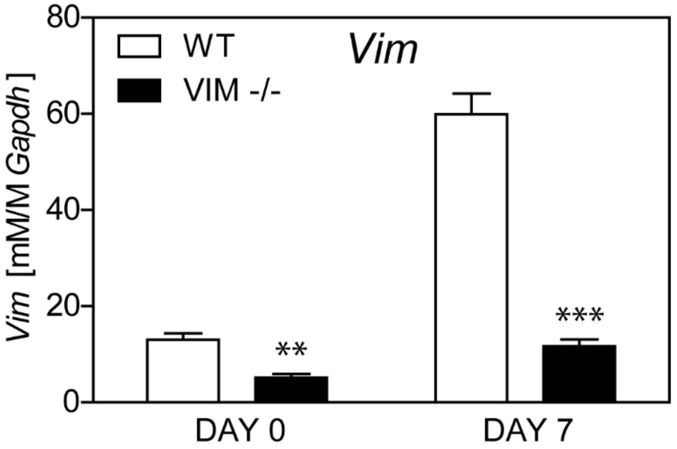

Supplementary Fig. S2. Vimentin gene expression is low in VIM -/- cells. At Day 0 VIM -/- ESCs have significantly lower vimentin gene expression (normalized to *Gapdh*) compared to WT ESCs. With 7 days of differentiation, vimentin gene expression in WT cells increases by >3x while VIM -/- cells remain markedly lower. Significant differences are indicated using asterisks (**p<0.01, ***p<0.001).

Supplementary Figure S3

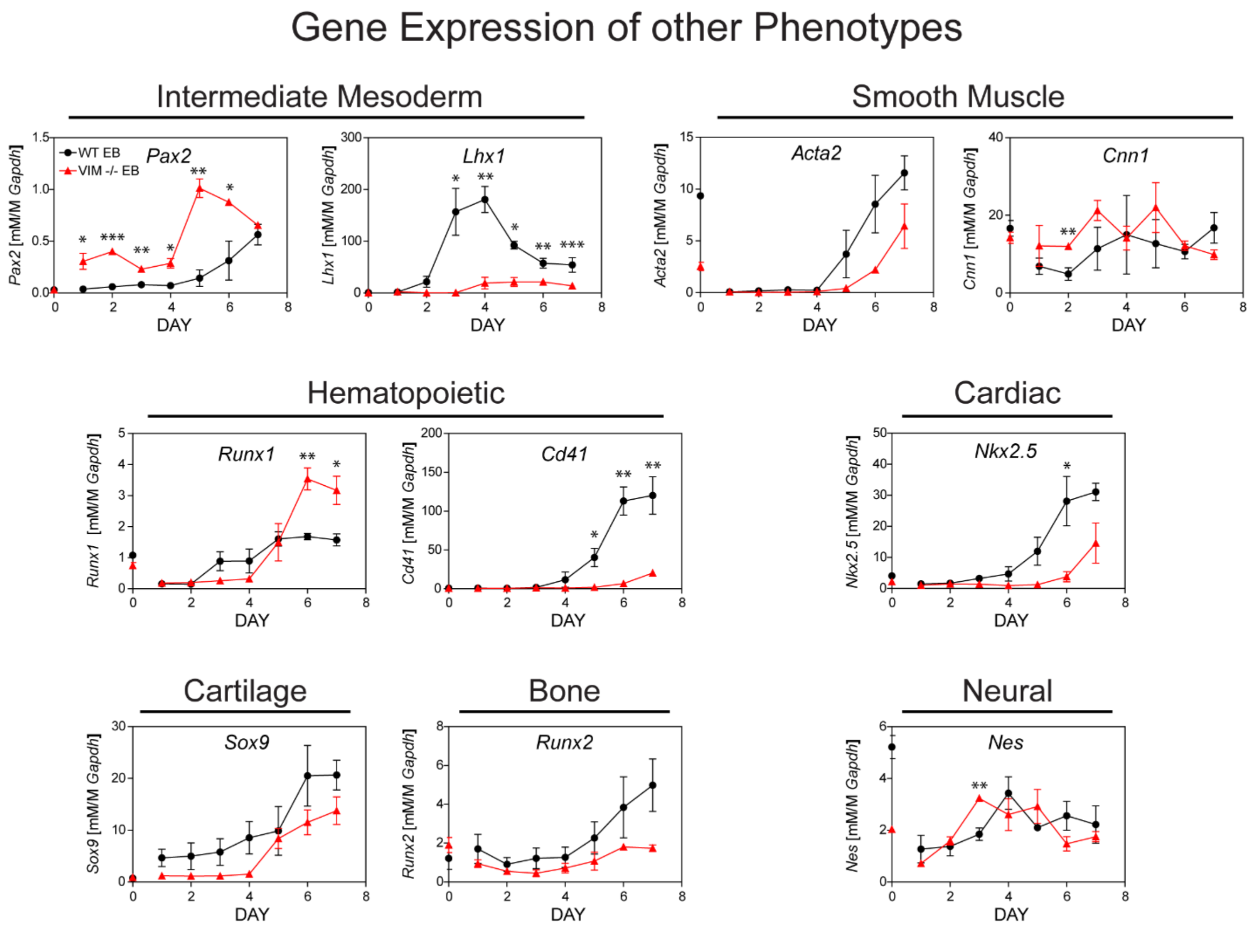

Supplementary Fig. S3. The absence of vimentin had no consistent effects on select markers of other phenotpes. Over 7 days of differentiation other mesodermal lineage specific genes were evaulated in WT EBs and VIM -/- EBs. Data are presented as mean ± SEM (n=3), with significant differences indicated using asterisks (*p<0.05, **p<0.01, ***p<0.001).

Supplementary Figure S4

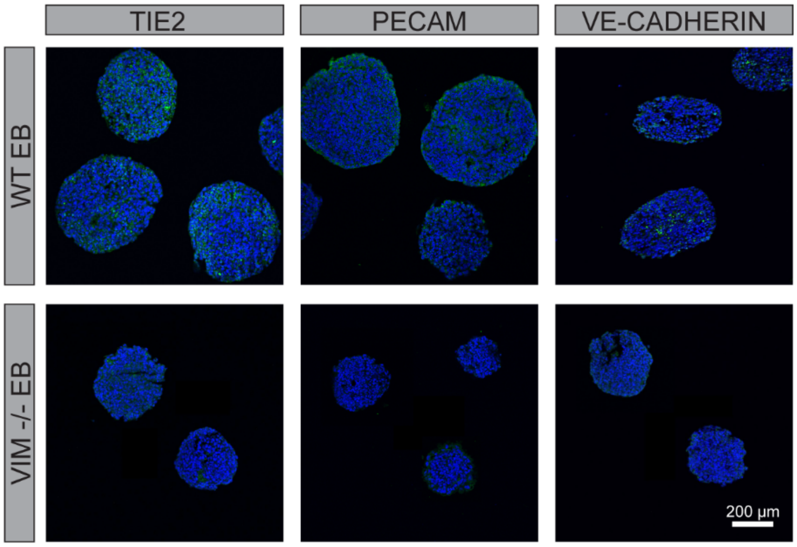

Supplementary Fig. S4. Immunohistochemistry analysis of endothelial markers in Day 6 WT EBs and VIM -/- EBs. Immunohistochemical analysis of TIE2, PECAM, and VE-CADHERIN protein expression (green) with a nuclear counterstain (blue) in EBs at Day 6. All images were taken at the same magnification and the scale bar represents 200 µm.

Supplementary Figure S5

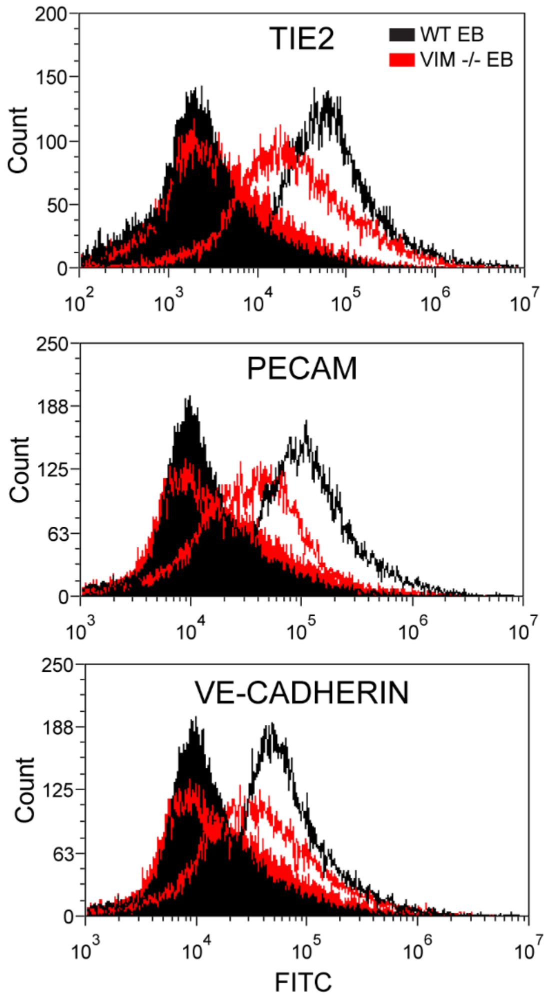

Supplementary Fig. S5. Endothelial differentiation is impaired in VIM -/- EBs compared to WT EBs. A representative histogram of TIE2, PECAM, and VE-CADHERIN protein expression is shown for WT EBs (black) and VIM -/- EBs (red), as well as their respective secondary antibody-only controls (shaded histograms).
